# Primed for Interactions: Investigating the Primed Substrate Channel of the Proteasome for Improved Molecular Engagement

**DOI:** 10.3390/molecules29143356

**Published:** 2024-07-17

**Authors:** Cody A. Loy, Darci J. Trader

**Affiliations:** Department of Pharmaceutical Sciences, University of California, Irvine, CA 92617, USA; loyc@uci.edu

**Keywords:** proteasome, inhibitor, substrate channel

## Abstract

Protein homeostasis is a tightly conserved process that is regulated through the ubiquitin proteasome system (UPS) in a ubiquitin-independent or ubiquitin-dependent manner. Over the past two decades, the proteasome has become an excellent therapeutic target through inhibition of the catalytic core particle, inhibition of subunits responsible for recognizing and binding ubiquitinated proteins, and more recently, through targeted protein degradation using proteolysis targeting chimeras (PROTACs). The majority of the developed inhibitors of the proteasome’s core particle rely on gaining selectivity through binding interactions within the unprimed substrate channel. Although this has allowed for selective inhibitors and chemical probes to be generated for the different proteasome isoforms, much remains unknown about the interactions that could be harnessed within the primed substrate channel to increase potency or selectivity. Herein, we discuss small molecules that interact with the primed substrate pocket and how their differences may give rise to altered activity. Taking advantage of additional interactions with the primed substrate pocket of the proteasome could allow for the generation of improved chemical tools for perturbing or monitoring proteasome activity.

## 1. Introduction to the Proteasome’s Structure

### 1.1. 20S Core Particle

**Figure 1:**
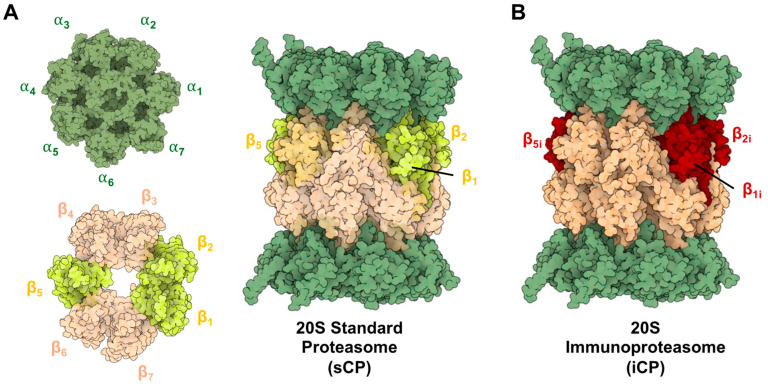
(**A**) Structure of the 20S Standard Core Particle (sCP), containing 14 distinct subunits repeated twice (28 total proteins), forming heptoheteromic rings that assemble into the active barrel-like structure. Catalytically active subunits are highlighted (yellow). (**B**) Structure of the 20S immunoproteasome (iCP) containing the same barrel-like assembly of subunits but with altered catalytic subunits (red). These isoforms are capable of degrading proteins that have been oxidatively damaged or unstructured, but their cleavage products will differ due to their altered substate specificities. PDB ID: 4R3O and 5LE5.

The proteasome is a barrel-like structure made by the incorporation of seven distinct types of α and β subunits in a tightly conserved and ordered manner [1,2]. The β subunits form two rings enclosing the catalytic chamber, with three displaying catalytic activity (β1—caspase-like, β2—trypsin-like, and β5—chymotrypsin-like) [3]. The α subunits form an additional two rings that cap either end of the complex, serving as gates for substrates to enter the inner catalytic chamber. When fully assembled, this proteasome is referred to as the 20S standard core particle (sCP) and is capable of functioning independently to degrade disordered and or misfolded proteins, Figure 1A [4,5,6,7,8,9,10].

When a cell experiences an inflammatory signal (such as exposure to interferon-γ or tumor necrosis factor-α) newly synthesized proteasomes begin to be expressed [11,12,13]. The newly synthesized immunoproteasomes (iCP) are assembled into the same barrel-like structure, but with the distinct difference that the catalytic subunits are swapped for “immuno”-subunits (β1i—chymotrypsin, β2i—trypsin, and β5i—chymotrypsin) that recognize protein substrates differently than their sCP counterpart, Figure 1B. This allows the iCP to preferentially generate peptide products with *C*-termini that more favorably interact with MHC-I molecules [14,15,16]. This is advantageous for cell surface antigen presentation to activate an immune response [17]. The iCP is also found to be expressed in varying amounts across several other disease types, such as cancer, leading to core particles that can be assembled into a variety of different combinations of catalytic β-subunits. The iCP is inducible in most tissue types and is constitutively expressed in cells of hematopoietic origin [18]. It is also important to note that “hybrid” isoforms also exist, which can be a combination of iCP and sCP subunits, depending on which isoforms are being actively assembled in the cell.

### 1.2. Ubiquitin Proteasome System Degradation

Although the sCP is capable of handling a fair amount of the protein load in cells, a different isoform of the proteasome (26S) is responsible for the degradation of proteins that have been tagged with polyubiquitin [19,20,21,22]. Ubiquitin is a small protein that serves as a signal for the proteasome to recognize for degradation. Prior to degradation, substrates are tagged with 4–5 units of ubiquitin by an E1-E2-E3 process that the 26S proteasome can recognize for degradation [23,24,25]. This process has recently been hijacked for therapeutic purposes with the development of PROTACs and ByeTACs that can increase the degradation of specific proteins [26,27,28].

**Figure 2:**
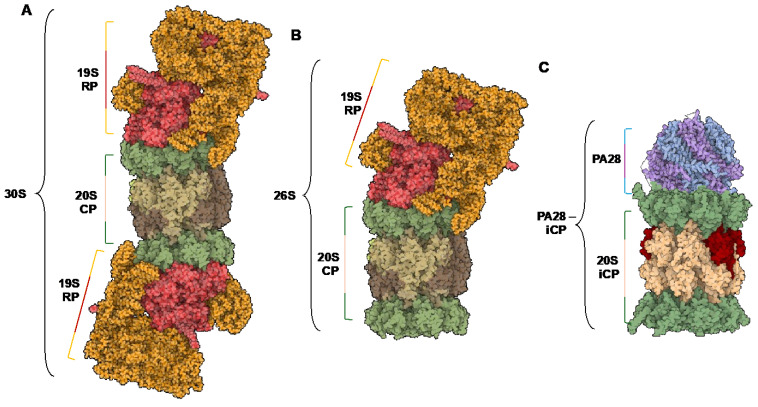
(**A**) Structure of the 30S isoform of the proteasome, containing a 20S catalytic core particle and two 19S regulatory particles. (**B**) Structure of the 26S isoform of the proteasome, containing a 20S catalytic core particle and one 19S regulatory particle. (**C**) Structure of the PA28—iCP complex. PDB IDs: 5GJR, 7DR6.

The 26S proteasome isoform consists of the catalytic barrel (20S sCP or iCP) and a 19S regulatory particle (19S RP) that can singly or doubly cap the complex. When capped with one 19S, the multicomplex is known as the 26S proteasome, and when doubly capped, it is referred to as the 30S proteasome [29,30], Figure 2A,B. The 19S RP is responsible for the recognition, deubiquitination, unfolding, and translocation of proteins into the catalytic chamber to be degraded [31,32,33,34,35,36]. This is facilitated through ATP-dependent and ATP-independent processes. As the protein substrate is shuttled through the catalytic chamber, the peptide’s amide bonds are hydrolyzed based on its amino acid sequence and the substrate specificities of the core particle (sCP, iCP, or hybrid CP).

### 1.3. Endogenous Activators

In addition to the 19S RP, there are other 20S activators in the cell that are capable of gate opening and increasing the rate of proteasome-mediated protein hydrolysis. The inflammatory stimuli required to signal the cell to begin synthesizing the iCP catalytic subunits can also alert the cell to begin expression of the proteasome activator subunits PSME1 and PSME2 (PA28α and PA28β, respectively) [37]. This ring-shaped heteromultimer has no enzymatic activity itself; however, it has been shown to activate the proteasome’s peptidase activity when binding to the alpha subunits, Figure 2C. It is still unclear whether this activation is due to the complex inducing an open gate confirmation that allows substrates to enter the catalytic chamber more rapidly or if it is acting as an allosteric activator of the catalytic subunits. Solving the PA28-iCP complex’s human isoform would greatly enhance our understanding of this mechanism; however, the bovine structure remains the only solved cryo-electron microscopy evidence of a partially opened gate [38]. Due to PA28’s subunits relying on the IRF1 gene to trigger its expression, it is most commonly associated with the iCP; however, since the sCP can still be present in the cell, PA28 is also capable of activating the standard isoform as well [39,40].

## 2. Structural Differences in Core Particles

### 2.1. Structural Overview

**Figure 3:**
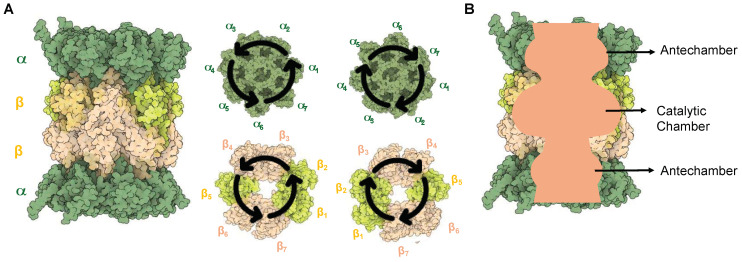
(**A**) Structure of the 20S sCP. The symmetry of the proteasome allows one set of α and β subunits to be clockwise, while the other two are counterclockwise in orientation. (**B**) A sliced view of the proteasome to show the two antechambers and the catalytic chamber.

To date, there have been numerous structural studies of the 20S proteasome that detail the arrangement of the subunits and modes of substrate recognition [1,41,42,43]. The 20S proteasome is a cylindrical barrel that consists of four rings—two containing the α-subunits (1–7) at either end of the complex and two containing β-subunits (1–7) that are arranged in a way that their N-termini face toward the inner chamber of the complex. Overall, the proteasome contains pseudo-C2 symmetry, with one set of α/β subunits running clockwise and the other counterclockwise, as shown in Figure 3A [44]. The inner cavity of the proteasome contains three compartments, two antechambers at either end of the complex enclosed by the α-subunits, and the catalytic chamber encompassed by all 14 β-subunits, Figure 3B [45,46,47]. The central catalytic chamber of the proteasome has been explored thoroughly; however, the antechamber functions still remain unclear. The 20S is capable of engaging with proteins in its “uncapped” isoform, leading to their degradation without the need for ubiquitin or an activator [48,49].

### 2.2. Substrate Channel Preferences

**Figure 4:**
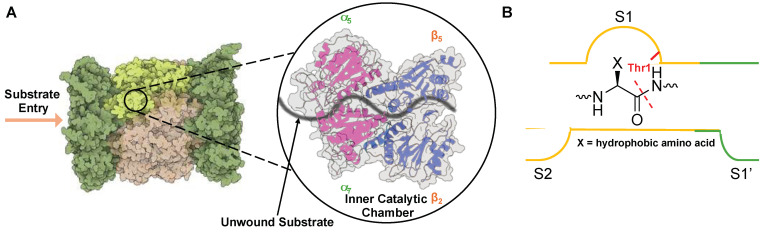
(**A**) Substrate entry into the 20S proteasome through the α-subunit portal into the inner catalytic chamber. (**B**) Active site hydrolysis of an unwound substrate by the β5 subunit. All subunits utilize an active site Thr (red) for hydrolysis of substrates but have altered substrate specificities due to differences in S1 and S2 substrate binding pockets.

As an unstructured protein enters the 20S core particle, it engages with three distinct catalytically active subunits (β1—caspase-like, β2—trypsin-like, and β5—chymotrypsin-like) that cleave after preferred amino acids, generating smaller peptide products that can be further digested into single amino acids by other cellular proteases [45,50,51]. The most active catalytic subunit, β5—chymotrypsin, cleaves preferentially after hydrophobic amino acids such as valine, phenylalanine, and tyrosine. This is due to the surface properties of the S1 substrate binding pocket in the non-primed channel, which houses the active Thr1 and interacts with amino acids in P1 upstream of the cleavage site, Figure 4. β1 contains a positively charged arginine, while β2 was identified to house a glycine and a negatively charged aspartate, leading to the ability to cleave after negatively charged (caspase-like) and positively charged (trypsin-like) residues, respectively [43,52,53].

Upon treatment with interferon-γ, the catalytic subunits incorporated into the standard proteasome are substituted with the corresponding immuno-subunits that have altered cleavage specificity [11,12]. These altered preferences are due to structural changes that have occurred in the unprimed substrate channel. The unprimed substrate channel is still the most well defined and characterized, with many studies done to understand the differences between the two isoforms. The β2i subunit remains identical to its sCP counterpart, except for the substitution of Asp53 (sCP) for Glu (iCP). The reason for this small change is still unclear, and additional roles that it may play have yet to be elucidated. In contrast to β2i, β1i has several important distinctions compared to its sCP counterpart. Several substitutions (T20V, T31F, R45L, and T52A) lead to an increase in hydrophobicity and shrink the size of the binding pocket for a protein substrate. These amino acid changes in the β1i subunit lead to the generation of peptide with small hydrophobic residues at the *C*-terminus, which improves the peptide’s ability to be loaded into a MHC-I complex [54,55,56].

**Figure 5:**
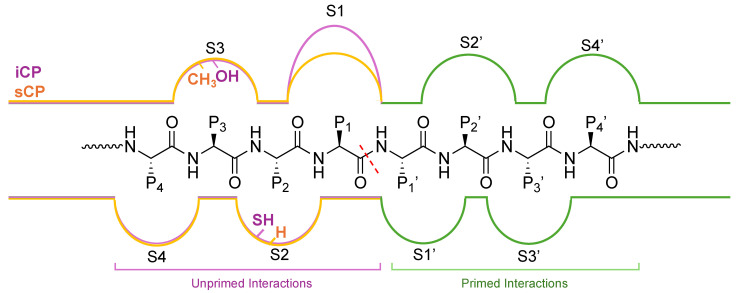
Substrate channel for the β5i (purple) and β5 (orange) subunits of the iCP and sCP, respectively. The unprimed channel has been thoroughly explored for differences in the substrate binding pockets S1–S4. The differences in substrate binding pockets S1′–S4′ have remained relatively understudied; however, several inhibitors and chemical probes have been found to take advantage of interactions in this channel to gain selectivity and potency. These indications hint that the primed substrate channel has interactions that can be harnessed when developing proteasome inhibitors or probes, and work is needed to further identify the crucial interactors.

The amino acids in the S1 pocket of the β5i and β5 are the same, except the S1 pocket of the β5i subunit is increased in size compared to β5, allowing bulky hydrophobic amino acids such as Trp to be recognized more efficiently. The S2 pocket makes the change Gly for a Cys or Ser, while the S3 pocket substitutes Ala (β5) by Ser (β5i), giving increased hydrophilic character, Figure 5.

The structure of the iCP only differs from the sCP in its substitution of the catalytic subunits β1i, β2i, and β5i. This similarity forces substrate affinity to rely solely on the interactions within the primed and unprimed substrate channels. The unprimed substrate channel has previously been explored in the section above and has key differences that allow substrate specificity to be achieved. The primed substrate channel, on the other hand, has remained less characterized and studied; however, there are a few key differences that can be noted. While the β2i subunit is identical to the β2 subunit, β1i is shortened by one residue compared to β1 [52]. Another key difference in the β5i channel is the substitutions of S115D and E116N that may affect substrate specificity and cleavage preferences. Although there seem to be only a few structural differences between the primed substrate channel and the unprimed channel, it has yet to be explored in detail how the interactions there can increase the selectivity and potency of binders. Taking advantage of key interactors in this channel could be of great interest to those seeking to generate improved inhibitors or chemical probes of the proteasome isoforms.

## 3. Rational Design of Small Molecule Proteasome Interactors

The ability of the cell to degrade proteins is a highly conserved and tightly regulated process regulated by the ubiquitin proteasome system (UPS). An impairment of this process can give rise to detrimental effects and several pathological conditions [57]. Cancer cells have been extensively studied in relation to disruption of the UPS, leading to elevated levels of stress, loss of cell cycle control, and increased protein accumulation. This has led to the exploitation of small molecules that bind to the proteasome’s catalytic subunits to inhibit its ability to degrade proteins, causing an accumulation of proteins and eventual apoptosis. The different substrate preferences between the sCP and the iCP have been explored to develop selective inhibitors for the two isoforms, with all FDA-approved proteasome inhibitors containing amino acids in P1–P5 that interact with the non-primed substrate channel specificity pockets S1–S4 [58].

### 3.1. Non-Primed Substrate Binders

#### 3.1.1. Boronic Acids

One of the most successful demonstrations of inhibiting the catalytic subunits of the proteasome was with the development of reversible di- and tri-peptidyl boronic acid inhibitors [59]. The development of Bortezomib (Velcade) by Millennium Pharmaceuticals, Inc. led to the first FDA-approved proteasome inhibitor of the sCP for the treatment of multiple myeloma in 2003 [60,61,62]. Bortezomib is a covalent, reversible binder to the sCP through a complex between the boronic acid and the catalytic Thr1 hydroxyl, leading to the formation of a tetrahedral adduct [59,63]. This interaction can also occur with the other two catalytic subunits, but to a much lesser extent [64,65]. Although Bortezomib excited the field and validated the proteasome’s core particle therapeutically, it has toxicity in non-target tissues, low bioavailability, resistance, and the required combination of other chemotherapies to increase potency [66].

Due to these limitations, several medicinal chemistry campaigns occurred to improve upon the current scaffold with second-generation inhibitors that had improved ADME properties [60]. The next FDA-approved boronic acid inhibitor, Ixazomib (2015), was the first sCP inhibitor to be given to patients orally [67]. Ixazomib belongs to the same structural class as Bortezomib, gaining selectivity through interactions in the non-primed channel with an alanine-leucine dipeptide core and a citrate-protected boric acid warhead [68]. This citrate ester allows Ixazomib to be administered orally [69].

#### 3.1.2. Epoxides

In the search for improved covalent inhibitors of the sCP and iCP, the natural product epoxomicin from actinomycete strain Q996-17 was identified to have antitumor activity [63]. Similarly to other inhibitors, epoxomicin covalently binds the catalytically active subunits; however, it is much more selective for the proteasome than other inhibitors by demonstrating little to no inhibition of other proteases [70,71]. This is believed to be due to the formation of a morpholino adduct created by a two-step process where Thr1 initially attacks the carbonyl carbon of epoxomicin. Next, the free amine can perform a nucleophilic attack on the C2 carbon of the epoxide, opening the ring and forming the morpholino product [72]. Since the proteasome is the only known protease to utilize an *N*-terminal Thr1, it is the only protease capable of forming this adduct with the epoxyketone inhibitors, which is believed to be the reason for their enhanced selectivity. Carfilzomib is another epoxyketone inhibitor that gained FDA approval in 2012. It has similar potency as Bortezomib; however, it is much more selective, leading to lessened side effects [60,73].

iCP-selective inhibitors have also been generated to treat diseases in which this proteasome isoform is overexpressed. ONX-0914 was the first reported selective inhibitor of the iCP subunit β5i [74]. It was developed through a medicinal chemistry campaign with the hopes of improving upon epoxomicin. ONX-0194 demonstrates 20–40 fold selectivity for the β5i subunit over the β5 counterpart [75]. From this scaffold, other iCP-selective inhibitors have been generated that have improved selectivity and potency [76,77]. Many other types of sCP and iCP inhibitors have been employed with a similar goal, such as vinyl-sulfones and aldehydes, and have been extensively described in other reviews, Table 1 [59,73,78].

### 3.2. Primed Substrate Binders

#### 3.2.1. Natural Products

**Figure 6:**
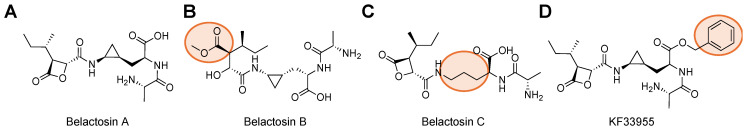
Structures of Belactosin A and its derivatives that engage with the primed substrate channel to inhibit proteasome activity. (**A**) Belactosin A contains a lactone ring to react with the 20S CP catalytic Thr. (**B**) Belactosin B does not have a lactone ring (orange circle) and is no longer effective at inhibiting 20S CP. (**C**) Belactosin C no longer has the *trans*-cycloproane ring but can still engage and inhibit 20S CP because of the lactone. (**D**) Optimization of Belactosin A led to improved inhibition with the introduction of the phenyl ring at the carboxylic acid. This demonstrates that there are moieties that can be explored to better engage with the primed substrate channel that lead to improved selectivity or potency.

Although the unprimed substrate channel has been extensively studied in the development of selective inhibitors for the proteasome isoforms, the primed substrate channel remains relatively understudied in regards to modifying proteasome activity with small molecules [99,100,101]. In 2000, a natural product isolated from *Streptomyces* sp. was found to inhibit proteasome activity and mediate cell cycle regulation through the cyclin/CDK pathway [92]. This natural product, named Belactosin A, possesses a β-lactone that covalently binds to the β5 subunit, resulting in opening the lactone ring and acylation of Thr1, Figure 6A [102]. To increase the potency of this class of inhibitors, other derivatives were synthesized, such as KF33955, which incorporates a benzl group; Belactosin B, which eliminates the β-lactone; and Belactosin C, which removes the *trans*-cyclopropane ring, Figure 6B–D [93,103,104]. The addition of KF33955’s benzyl group to Belactosin A’s carboxylic acid indicates there is some preference for hydrophobic interactions within the primed substrate channel to increase its reactivity. It also neutralizes the scaffold, aiding in permeability and adding hydrophobicity.

**Figure 7:**
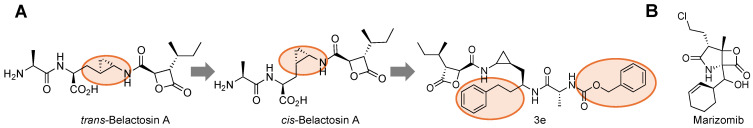
(**A**) SAR of Belactosin A from its *trans*-isomer to *cis*- was found to increase its IC_50_ value. Further optimization of the scaffold led to the development of compound **3e**, which has toxicity similar to that of Bortezomib, all through interactions, highlighted in orange, in the primed substrate channel. (**B**) Structure of Marizomib that interacts with all catalytic subunits of the 20S CP and is orally available.

Structural studies performed with Belactosin and yeast proteasomes found that it bound primarily in the primed substrate pocket [53]. In addition, other groups have been able to perform Structure Activity Relationship (SAR) on this class of compounds to increase its potency by synthesizing unnatural analogs that switch the *trans*-cyclopropane group to its *cis*-orientation [105]. Further optimization allowed for more interactions with the primed substrate channel, leading to the development of the 3e derivative that had IC_50_ values comparable to bortezomib for the β5 subunit, Figure 7A [103]. The *cis*-cyclopropane isomer also inhibited both β5 and β5i, indicating isoform selectivity may not be achieved through primed interactions alone.

In addition to the Belactosins, another natural product, the β-lactone inhibitor from the marine actinomycete *Salinispora tropica*, is the only non-peptide proteasome inhibitor used for multiple myeloma and gliomas, Figure 7B [106]. Marizomib elicits its effects by interacting through its carbonyl with Thr1’s hydroxyl irreversibly. As such, it is able to inhibit all three catalytic subunits of the standard proteasome for long periods of time [73]. Unlike Bortezomib, marizomib is orally available and has been shown to be effective against Bortezomib-resistant cell lines [80]. This recently underwent several phases of clinical trials for glioblastomas [107,108]. This class of molecules has shown that there are potential interactions that are important to consider when designing SAR studies for proteasome inhibitors in the primed substrate channel.

#### 3.2.2. Peptide Based Interactors

**Figure 8:**
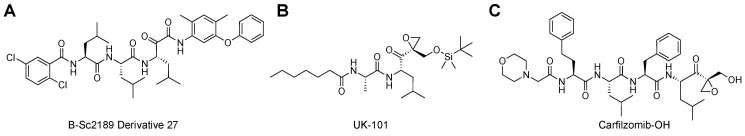
(**A**) α-ketoamide was developed to engage the primed substrate channel to increase selectivity. SAR derivative 27 was the most potent and selective of the inhibitors developed, with IC_50_ values in low nM concentrations as well as over 4-fold selectivity for the sCP over iCP. (**B**) UK-101 was developed to gain selectivity for the LMP2 subunit of the iCP by engaging with the primed substrate pocket. (**C**) Derivatives of the FDA-approved proteasome inhibitor Carfilzomib have been developed to overcome resistance seen with current proteasome inhibitors. By adding interactions to the primed substrate channel, this derivative increased its potency and was able to be effective against proteasome inhibitor resistance cells.

Beyond natural product inhibitors that take advantage of the primed substrate channel, work done by Schmidt [109,110,111] and Groll [98] shows that SAR of proteasome inhibitors into the primed substrate channel not only improves inhibitory performance but can also create selectivity differences for the two isoforms. These molecules, α-ketoamides, are shown to be highly active reversible inhibitors of the proteasome isoforms [111,112]. From the initial efforts of this work, refined screens were performed to try and improve the targeting towards β5 by altering the interactions in the S1′ pocket [109]. The researchers found that alterations made to the groups interacting with the primed substrate channel led to differences in toxicity profiles and isoform selectivity. These variations led to the most potent compound of their series using a 3-phenoxy-4,6-dimethylphenyl ketoamide (IC_50_ = 23 nM) with 4.2-fold selectivity over β5i, Figure 8A. This was further confirmed with molecular modeling of the compound in the substrate channel, elucidating that it does interact with the S1′ pocket of the sCP but is not as engaged with the S1′ pocket of the iCP.

UK-101 was developed to test the feasibility of inhibiting other catalytic subunits beyond the well-established β5, Figure 8B. Developing inhibitors for the iCP is of great interest, as they are typically expressed in areas of inflammation or diseased cells. This could provide increased selectivity and minimize the side-effects seen with current proteasome inhibition therapies [77,78]. LMP2 (β1i) is of interest as it has been shown to be overexpressed in neurodegenerative brains, such as those with Alzheimer’s and Huntington’s disease, as well as cancers like multiple myeloma [113,114,115]. This work set out to explore whether derivatizing a known inhibitor of β5i/β1i (dihydroeponemycin) at its P1′ position could lead to selective inhibition of only β1i [116]. By screening different groups in this position, they were able to identify their lead compound, UK-101, that contained a TBDMS group that was shown to selectively inhibit the LMP2 subunit, allowing researchers to better understand its therapeutic relevancy and further validating the primed substrate channel as an important interactor.

Carfilzomib, a peptide-based epoxyketone that was developed to improve upon the downfalls of Bortezomib, has been a great option for proteasome inhibition therapy [117,118,119]. However, this has led to a rise in cross-resistance, and researchers have begun exploring alternative routes to overcome it. One method that has been exploited is by identifying differences that can be harnessed in the primed substrate channel that are not being utilized by current proteasome inhibitors. Lee et al. were inspired by the development of UK-101 and its prime channel interactions and hypothesized that Carfilzomib could be modified to better engage with P1′ to overcome its resistance [97]. This group reported that altering the P1′ interactor to a small polar moiety led to 10-fold improved potency compared to the parent drug, and a 3-fold increase in potency for resistant cell lines, Figure 8C [97]. These all help validate the primed channel as therapeutically relevant and should be further explored to see how it can be utilized when attempting to discover proteasome isoform-selective inhibitors.

### 3.3. Primed Substrate Chemical Probe Interactors

**Figure 9:**
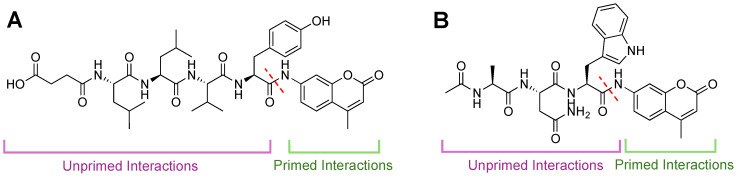
Fluorescent activity-based AMC probes for 20S CP. Intact probes are non-fluorescent until liberated by cleavage from the proteasome. Specificity has generally been achieved through unprimed substrate interactions (purple). As substrate (green) is cleaved (dashed red line), AMC is released, and fluorescence increases are monitored over time. (**A**) Structure of Suc-LLVY-AMC, which is selective for the chymotrypsin-like activity of the β5 subunit. (**B**) Structure of Ac-ANW-AMC, which is selective for the chymotrypsin-like activity of the β5i subunit.

Beyond looking at inhibitors of the proteasome isoforms, there are other small molecules or probes whose activity could be dependent on engaging with the primed substrate channel. Monitoring the proteasome’s activity with activity- and inhibitor-based probes has been beneficial for understanding this dynamic enzyme complex [120,121]. Most activity-based probes contain a peptide recognition sequence for the unprimed substrate channel, conjugated to a fluorophore such as 7-amino-methylcoumarin (AMC), Figure 9A,B. The fluorophore moiety of the probe must engage with the unprimed substrate channel, and this engagement is mostly ignored during probe design [122,123,124,125].

**Figure 10:**
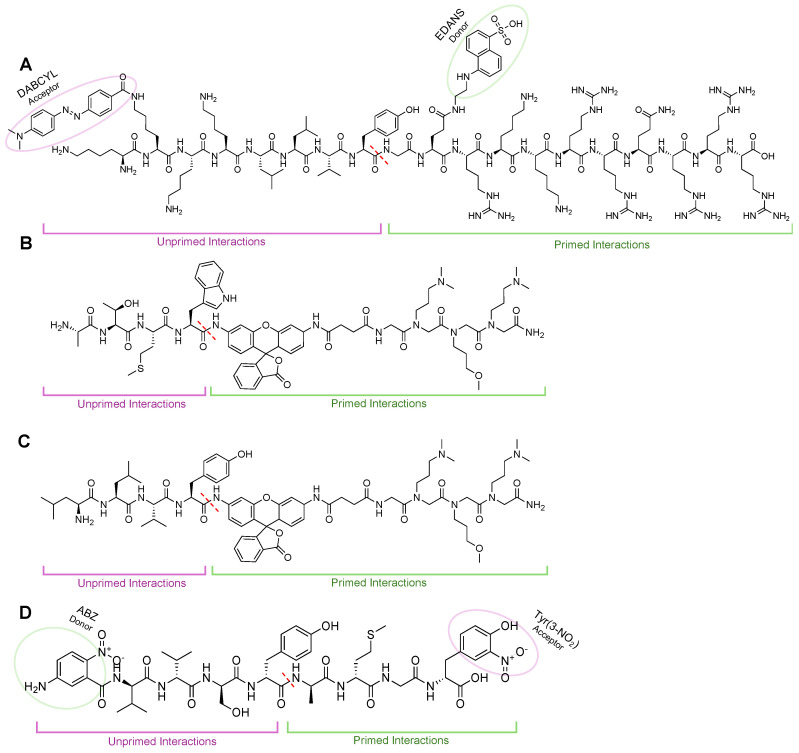
(**A**) TED FRET reporter to monitor sCP cleavage activity. When intact, FRET reporter signal for EDANS is quenched by the acceptor, DABCYL. Upon interaction with the 20S sCP, the bond between phenylalanine and alanine is cleaved (dashed red line), leading to an increase in fluorescent signal from liberated EDANS. (**B**) Rhodamine-based probes to monitor iCP activity biochemically or in cells (TBZ-1). iCP recognition sequence ATMW conjugates a rhodamine-peptoid that is non-fluorescent until interaction with the β5i subunit to cleave the bond between Trp and rhodamine (red-dashed line) that allows for increase in fluorescent signal to be monitored over time. (**C**) Rhodamine-based probes to monitor sCP activity biochemically or in cells (TAS-1). The sCP recognition sequence LLVY conjugates a rhodamine-peptoid that is non-fluorescent until interaction with the β5 subunit to cleave the bond between Tyr and rhodamine (red-dashed line), which allows for increase in fluorescent signal to be monitored over time. (**D**) FRET probe generated through combinatorial library for primed substrate interactors. Primed interactions led to probes being selective for iCP, demonstrating that engagements in this channel can lead to more selective probes/inhibitors.

AMC probes are mainly reliable in cell lysates or when utilizing purified proteasomes. Our group and others set out to develop cell permeable activity-based probes to monitor proteasome activity in real time. Several Foerster resonance energy transfer (FRET) peptides to monitor proteasome activity have been developed [126,127]. Both have included the DABCYL-EDANS FRET moieties. One FRET peptide was comprised of 8-amino acids between the DABCYL and EDANS groups, with the cleavage region in the center of the peptide. The TED peptide (Tat-Edans-Dabcyl) reporter contains a 10-mer amino acid sequence from the transfer domain of the Tat protein to aid in permeability and uses the same FRET pair, Figure 10A [128,129]. In both cases of the FRET activity probes, significant engagement of the primed substrate channel must be utilized. These probes demonstrated that the proteasome could engage with large unnatural substrates beyond AMC in its primed substrate channel.

To further improve activity-based probe design, two peptide/peptoid hybrids were also generated by the Trader lab that contained a rhodamine fluorophore that could be used in cell-based assays, Figure 10B,C [130,131,132]. Using a one-bead-one-compound library, the researchers were also able to develop a selective immunoproteasome probe, something that was unavailable at the time [131]. These probes were validated by fluorescent plate reader assays and confocal microscopy and required the peptoid tail for adequate cleavage of rhodamine, yet how this was interacting with the primed substrate channel has still not been investigated. Other groups have also tried to generate cell permeable activity-based probes similarly [133].

These examples have demonstrated that the primed substrate channel can play a role in the ability to uncage probes. The AMC/rhodamine moieties are adjacent to the scissile amide bond and are widely different in size and chemical structure. The EDANS from the FRET probe extend further into the channel and have more primed peptide interactions than the AMC/rhodamine probes. There is potential for the S1′ and S2′ pockets to preferentially or non-preferentially interact with the different cargo, yet this has never been explored as most assays only rely on relative fluorescence output.

Gruba et al. set out to demonstrate how the primed channel can be harnessed when developing chemical tools to monitor proteasome isoform activity, as it strongly affects substrate cleavage efficacy [134,135]. In this study, the researchers utilized a quenched reporter that contained a 5-amino-2-nitrobenzoic acid (ANB) and 3-nitrotyrosine (Tyr(3-NO_2_)) donor-acceptor pair. The scaffold for the reporter utilized ABZ-Val-Val-Ser-Tyr-Ala-X_2_′-X_3_′-Tyr(3-NO_2_)-NH_2,_ where X’s are amino acids that would interact with the prime substrate channel produced by a combinatorial library. Upon incubation with human proteasome isoforms, it was found that the sequence ABZ-Val-Val-Ser-Tyr-Ala-Met-Gly-Tyr(3-NO_2_)-NH_2_ was most susceptible to iCP cleavage, Figure 10D. This demonstrates that there are key interactions that are not being considered in the current probe and inhibitor design that could potentially improve the selectivity and activity of the binders. The interaction with the unprimed substrate channel was further validated by molecular modeling and testing in human cancer samples compared to healthy individuals [134].

Beyond cleavable probes, others have generated activity-based probes that irreversibly bind the proteasome’s active site through enzyme-catalyzed reactions. These types of probes generally consist of a reactive group, a recognition sequence, and a reporter such as a fluorophore [136]. These types of probes can be beneficial for studying cells when other probes are not cell permeable. The recognition element can be modified to allow for selective reactions with the different subunits of the proteasome or its various isoforms. The majority of the fluorophores developed rely on the epoxyketone or vinyl-sulfone warhead to covalently bind the subunit of interest [137,138]. The fluorescent tag can vary depending on the desired use; however, the BODIPY fluorophore is a common choice. With these probes, most interactions occur within the non-primed substrate channel to gain selectivity for the subunit of interest; however, the tail connecting to the fluorophore can move around freely. This could give rise to potential applications where taking advantage of the primed substrate channel may be beneficial. It has yet to be investigated how activity-based probes like these can be used to monitor the primed channel, but there are interactions discussed here that could lead to the development of newly designed probes for the primed channel that could help researchers gain further insight.

A recent study done by our group has started to investigate the preferences of primed substrate interactions [139]. The sCP recognition sequence LLVY was appended to a variety of different amino acids to determine what amino acid was the most effectively cleaved at this position. The various peptides were incubated with a purified 20S proteasome, and cleavage rates were investigated using LC/MS analysis. From this small dataset, it was determined that polar and/or hydrogen bonding R groups in the meta position of aromatic rings are favored by the S1′ binding pocket. It was also determined that the hydrolysis did not rely on the electrophilicity of the substrates amide bond, suggesting that the hydrolysis rate may be more dependent on substrate engagement and orientation. This was one of the first studies performed, looking specifically at what preferences could be harnessed in the sCP-primed substrate channel. Performing a similar study with the iCP would also be of interest in determining if there are more preferences that could be harnessed for isoform selectivity between the two in the primed channels.

## 4. Conclusions

In summary, the proteasome is a complex enzyme that is essential for cells to survive and tightly regulates protein levels to maintain homeostasis. As such, the proteasome has become an excellent target for inhibitors that can disrupt this balance, leading to the apoptosis of a diseased cell highly dependent on its activity to survive. In addition, a variety of probes have been developed to better monitor the different isoforms and various catalytic subunits and better understand their role in disease models. The design of these inhibitors and probes generally relies on binding interactions within the unprimed substrate channel of the catalytic subunits. This is because this substrate channel has been extensively studied and its potential binders are well established. In contrast, the primed substrate channel remains understudied in terms of how its interactions could be harnessed to develop improved inhibitors or chemical probes. There have been several reports trying to advance the field in this direction with a few inhibitors that have been identified to gain preference and increase potency by engaging in the primed channel. In addition, the probe field has also started to explore what interactions can be utilized to gain selectivity by the primed substrate channel alone. This information will be important as researchers continue to develop therapeutics for the proteasome. The additional interactions with the primed substrate channel can be used in cells that have acquired resistance to traditional unprimed substrate inhibition. Harnessing the primed substrate channel could allow for more information to be learned about this complex system and unlock new therapeutic options.

## Figures and Tables

**Table 1:** Proteasome and immunoproteasome inhibitors and their corresponding warheads, substrate interaction, and subunit specificity.

Name	Class	Substrate Channel	Subunit	Stage	Ref
Bortezomib	Boronic Acid	Non-primed	β5/β1	FDA Approved	[65,79]
Ixazomib	Boronic Acid	Non-primed	β5	FDA Approved	[80]
Delanzomib	Boronic Acid	Non-primed	β5	Clinical	[81]
MG-132	Aldehyde	Non-primed	β5	Research Tool	[82]
PSI	Aldehyde	Non-primed	β5 β2, β1	Pre-Clinical	[59]
Felutamide B	Aldehyde	Non-primed	β5, β2, β1	Pre-Clinical	[83]
CEP1612	Aldehyde	Non-primed	β5	Pre-Clinical	[84]
ISPI-001	Aldehyde	Non-primed	β5i/β1i	Pre-Clinical	[77]
Epoxomicin	Epoxide	Non-primed	β5	Research Tool	[71]
Carfilzomib	Epoxide	Non-primed	β5	FDA Approved	[85]
Oprozomib	Epoxide	Non-primed	β5, β5i	Clinical	[86]
ONX-0914	Epoxide	Non-primed	β5i	Research Tool	[74]
PR-924	Epoxide	Non-primed	β5i	Pre-Clinical	[87]
KZR-616	Epoxide	Non-primed	β5i	Clinical	[76]
LU-002i	Epoxide	Non-primed	β2i	Pre-Clinical	[88]
NLVS	Vinyl-Sulfone	Non-primed	β5	Pre-Clinical	[89]
HMB-Val-Ser-Leu-VE	Vinyl-Sulfone	Non-primed	β2	Pre-Clinical	[90]
Z-NH-(CH_2_)_5_-CO-Leu-Leu-Leu-VE	Vinyl-Sulfone	Non-primed	β1	Research Tool	[91]
Belactosin A	β-lactone	Primed	β5	Pre-Clinical	[92]
Belactosin C	β-lactone	Primed	β5	Pre-Clinical	[53]
KF33955	β-lactone	Primed	β5	Pre-Clinical	[93]
Marizomib	β-lactone	Primed	β5, β2, β1	Clinical	[73,94]
Omuralide	β-lactone	Primed	β5	Pre-Clinical	[92,95]
UK-101	Epoxide	Primed	β1i	Pre-Clinical	[96]
Carfilzomib-P1′	Epoxide	Primed	β5	Pre-Clinical	[97]
B-Sc2189	α-ketoamide	Primed	β5	Pre-Clinical	[98]
